# Age-dependent normal values for the ‘Infant Gastroesophageal Reflux Questionnaire Revised’

**DOI:** 10.1007/s00431-023-05281-w

**Published:** 2023-11-06

**Authors:** Marinde van Lennep, Fréderique Lansink, Marc A. Benninga, Michiel P. van Wijk

**Affiliations:** 1 Amsterdam UMC location University of Amsterdam, Department of Pediatric Gastroenterology and Nutrition, Emma Children’s Hospital, Meibergdreef 9, H7-292, PO Box 22700, 1100 DD, Amsterdam, The Netherlands; 2 Department of General Internal Medicine, University Hospital Leuven, Leuven, Belgium; 3 Amsterdam UMC location Vrije Universiteit Amsterdam, Department of Pediatric Gastroenterology, Emma Children’s Hospital, Boelelaan 1117, Amsterdam, The Netherlands

**Keywords:** Infant, Gastroesophageal reflux, Gastroesophageal reflux disease, Questionnaire

## Abstract

The Infant Gastroesophageal Reflux Questionnaire Revised (I-GERQ-R) is used to score and evaluate gastroesophageal reflux (GER) related symptoms such as regurgitation, irritability and crying. A cut-off of ≥ 16 has been suggested to discriminate between GER and GER-disease. However, age-specific normal values were not assessed. Aim of this study was to determine age-specific normal values in healthy infants. Cross-sectional survey in healthy infants aged 0–24 months during their regular check-ups at well-baby clinics and through an online questionnaire using the I-GERQ-R. Infants with a history of (suspected) GER-disease were excluded. Spearman’s correlation coefficient was calculated to explore age-related trends. A total of 979 healthy infants (47% male, median age 6 [0–24] months) were included. Median I-GERQ-R score, regardless of age, was 6 (range: 0–27). I-GERQ-R scores significantly decreased with age (*r*_*s*_ = -0.569, p < 0.001). Of all infants, 49 (5%) had a score of ≥ 16 which previously has been considered ‘suggestive of GER-disease’. Scores of ≥ 16 were most frequently seen in newborns aged 0–4 months (16% in 0–1 months to 4% in 3–4 months) and disappeared after the age of 16 months. High scores in the first months of life were caused by a high prevalence of regurgitation, colic-associated symptoms and hiccup frequency.

*Conclusion*: Age-dependent normal values for the I-GERQ-R show that reflux symptoms decrease in the first 24 months of life in healthy infants. Scores of ≥ 16 should not necessarily be considered abnormal in young infants. Validation as a diagnostic tool for GER-disease using age-appropriate normal values is needed.

**Table:** 

**What is Known:** *• The Infant Gastro Esophageal Reflux Questionnaire Revised (I-GERQ-R) was developed to objectively score and evaluate GER-related symptoms in infants.* *• Previously a cut off of ≥ 16 has been suggested to differentiate between gastro esophageal reflux (GER) and GER-disease.*	
**What is New:** *• We present age-specific normal values of the I-GERQ-R in healthy infants.* *• I-GERQ-R scores clearly decrease with age and up to 16% of young healthy infants have a score that is above the previously suggested cut-off score of 16 for GER-disease.*

**Supplementary Information:**

The online version contains supplementary material available at 10.1007/s00431-023-05281-w.

## Introduction

In infants, symptoms of gastroesophageal reflux disease (GER-disease) include regurgitation, irritability, crying, and feeding refusal [1–3]. These symptoms are nonspecific, as they are also common in healthy infants and overlap with symptoms of cow’s milk allergy or infant colic.

The Infant GastroEsophageal Reflux Questionnaire Revised (I-GERQ-R), a 12-item multiple-choice questionnaire, was developed to objectively score and evaluate gastroesophageal reflux (GER) related symptoms in infants such as regurgitation, irritability and crying [4]. Previously, a cut off of ≥ 16 points has been suggested to differentiate between healthy infants and those with GER-disease [4]. The I-GERQ-R has however not been validated to discriminate between infants with GER-disease and symptomatic infants *without* GER-disease. Additionally, potential age-related changes in I-GERQ-R scores were not taken into account [4]. The aim of this study was to determine age-specific normal values for the I-GERQ-R in healthy infants (0–24 months).

## Methods

This study is a cross-sectional survey regarding GER related symptoms in healthy infants aged 0–24 months.

### Ethical approval

The study was exempted from official approval by our local institutional review board (W19_291#19.344) as the medical research involving human subjects act did not apply.

### Study population and recruitment of subjects

Healthy infants (0–24 months) visiting well baby clinics in the greater Amsterdam area between October 2017 and March 2020 were eligible for the study. Additionally, healthy infants that were recruited for a previous study [5] at the same well baby clinics between February and October 2015 were included. Parents or caregivers of healthy infants were approached during their regular check-ups and their infants were checked for exclusion criteria. Healthy infants were additionally recruited through online advertisements until June 2021. Parents that expressed their interest online through a GCP clinical database (Castor Edc) were contacted and if infants did not fulfil exclusion criteria, the I-GERQ-R was surveyed.

### Exclusion criteria:

Infants were excluded when

- parents or care givers had poor understanding of Dutch or English language;
- gestational age at birth < 34 weeks;
- current GER symptoms (i.e. excessive crying, regurgitation, poor growth or weight loss) being experienced as ‘moderate’, ‘severe’ or ‘very severe’ by parent/caregiver.
- any health care professional had been consulted at any time before screening, regarding possible GER-related problems, such as excessive crying, regurgitation, poor growth or weight loss.
- infants used pharmacological and/or non-pharmacological GER therapies either at screening or before;

### Questionnaires

#### I-GERQ-R [6]

The I-GERQ-R is a 12 item multiple choice questionnaire in which frequency and quantity of GER symptoms in the past 7 days are surveyed [4]. It consists of subscales regarding regurgitation related symptoms and colic related symptoms. A maximum total score of 42 can be achieved. Higher scores point towards a larger burden of GER symptoms.

#### Clinical survey

A short 4-item clinical survey regarding the child’s current age (months), gestational age and reflux-related history was developed to screen eligible infants for exclusion criteria (see Supplemental file 1).

### Statistical analysis

SPSS (IBM Statistical Package for the Social Sciences [SPSS] for Windows, v 26.0 Armonk, NY: IBM Corp) was used for calculation of age-dependent I-GERQ-R scores, regurgitation and colic-related subscores and for analysis of median and P5-95 scores of I-GERQ-R scores.

Spearman’s correlation coefficient (*r*_*s*_) was calculated to explore age-related trends. Spearman’s correlation was also used in order to assess whether there was an association between regurgitation subscores and colic subscores. Strength of the correlation was classified as: *r*_*s*_ = 0.00–0.19 “very weak”; *r*_*s*_ = 0.20–0.39 “weak”; *r*_*s*_ = 0.40–0.59 “moderate”; *r*_*s*_ = 0.60–0.79 “strong”; *r*_*s*_ = 0.80–1.0 “very strong”.

## Results

One thousand and four infants were screened for eligibility. Six hundred fifty-five (65.2%) were included at well baby clinics and 349 (34.8%) were recruited through the online advertisement. Twenty-five infants (2.49%) were excluded from analysis because of ex-prematurity (n = 5), consultation of a health care professional for possible GER-related problems (n = 17), or because caregivers considered their child’s GER symptoms as moderate to very severe (n = 4, of which n = 1 also consulted a health care professional for GER symptoms).

A total of 979 infants (47% male, median age 6 [0–24] months) were included in this study. Median I-GERQ-R score of all infants, regardless of age, was 6 (range: 0–27). Of all infants, 49 (5%) had a score of ≥ 16 which is currently considered ‘suggestive of GER-disease’ [4].

### Correlations of total I-GERQ-R scores with age

Total I-GERQ-R scores, regurgitation- and colic-related subscores, correlated moderately with age (*r*_*s*_ = -0.569, *p* < 0.001; *r*_*s*_ = -0.554, *p* < 0.001 and *r*_*s*_ = -0.380; *p* < 0.001 respectively) and are displayed in Table 1.

**Table 1:** I-GERQ-R scores

	**Age (months) and number of patients**											
	**0–2** **(n = 198)**	**2–4** **(n = 157)**	**4–6** **(n = 129)**	**6–8** **(n = 115)**	**8–10** **(n = 81)**	**10–12** **(n = 59)**	**12–14** **(n = 52)**	**14–16** **(n = 61)**	**16–18** **(n = 36)**	**18–20** **(n = 37)**	**20–22** **(n = 23)**	**22–24** **(n = 31)**
**Total I-GERQ-R score**	10 (0–24)	9 (0–27)	7 (0–26)	6 (0–18)	5 (0–20)	4 (0–17)	4 (0–22)	3 (0–16)	3 (0–11)	4 (0–14)	2 (0–8)	2 (0–12)
**Regurgitation subscore**	3 (0–8)	3 (0–9)	2 (0–7)	2 (1–8)	1 (0–6)	0 (0–3)	0 (0–6)	0 (0–5)	0 (0–5)	0 (0–3)	0 (0–4)	0 (0–4)
**Colic associated subscore**	4 (0–11)	3 (0–11)	2 (0–8)	1 (0–11)	2 (0–9)	1 (0–12)	1 (0–9)	1 (0–5)	1 (0–7)	2 (0–10)	1 (0–6)	1 (0–7)

Median and range of total I-GERQ-R scores and regurgitation- and colic associated subscores

**Fig. 1:**
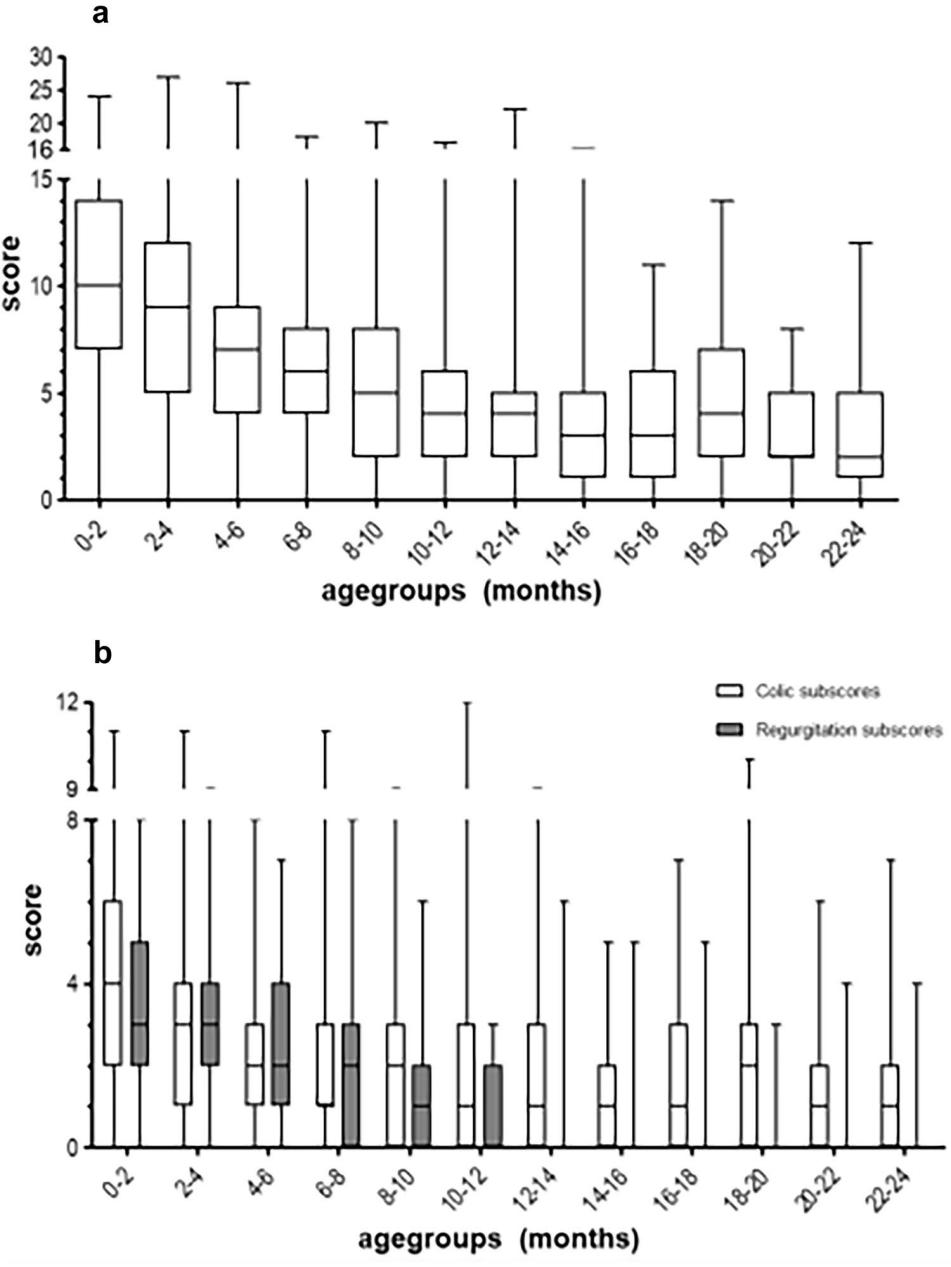
**a** Median and ranges of total I-GERQ-R scores. Boxes show interquartile ranges; whiskers show range; **b** Median and ranges of I-GERQ-R subscores scores. Boxes show interquartile ranges; whiskers show range

**Fig. 2:**
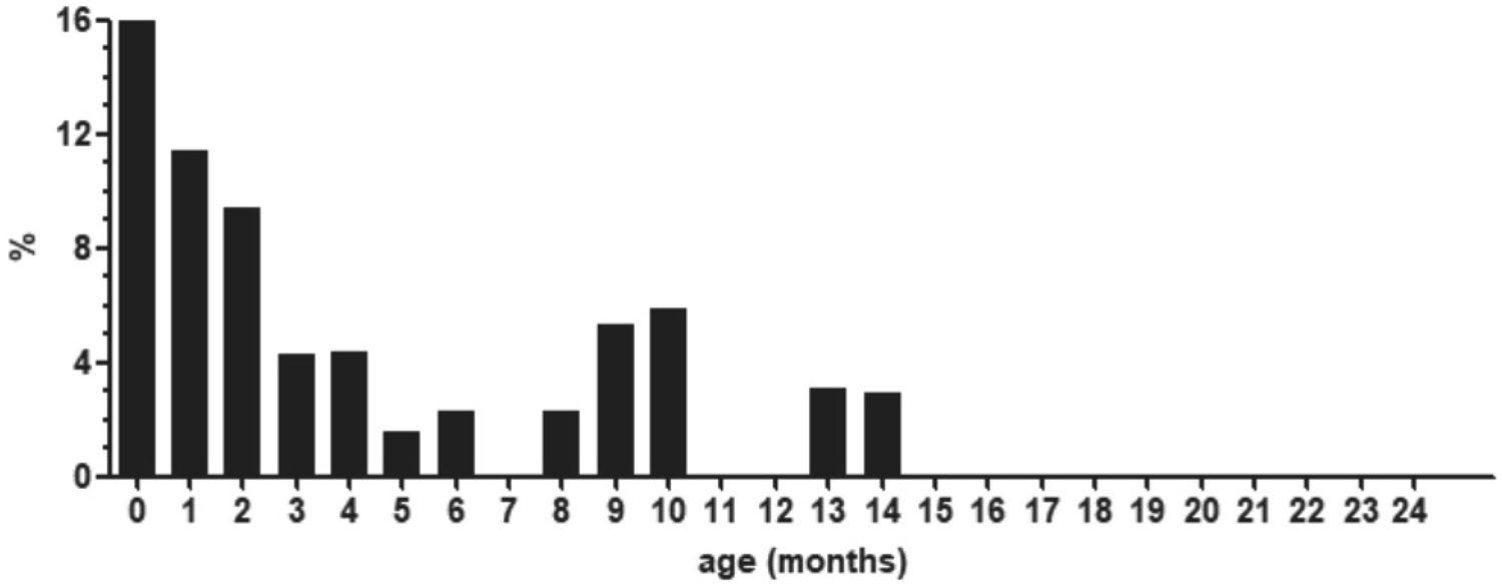
Percentage of healthy infants with a total I-GERQ-R score of ≥ 16, which is currently considered ‘suggestive of GERD’

Total I-GERQ-R scores (Fig. 1a), as well as regurgitation- and colic-related subscores (Fig. 1b) significantly decreased with age. A total score of ≥ 16 was most often seen in newborns aged 0–1 month (12/75, 16%). The percentage of cases with scores ≥ 16 rapidly decreased to 4% (3/68) at the age of 4–5 months and disappeared after the age of 15 months (see Fig. 2).

### Correlations of separate I-GERQ-R items with age

When looking at all I-GERQ-R questions separately, regurgitation frequency, regurgitation quantity and hiccup frequency showed moderate and significant negative correlations with age (*r*_*s*_ = -0.517, *p* < 0.001; *r*_*s*_ = -0. 488, *p* < 0.001 and *r*_*s*_ = -0.593; *p* < 0.001 respectively, see Table 2). The items ‘stopping with eating soon after starting the meal’ and ‘more than usual crying or fussing’ did not show any correlation with age, the remaining I-GERQ-R items showed low or very low correlations with age (Table 2). ‘Feeding refusal’ was the only I-GERQ-R item with a (very weak) *positive* correlation with age and was most frequently reported in infants aged 15 months and older.

**Table 2:** Correlation between I-GERQ-R questions and age (in months)

**Question**	**Spearman’s rho**	**Effect size**	***P*** **value**
1. During the past week, how often did the baby usually spit-up (anything coming out of the mouth) during a 24-h period?	**-0.517**	**Moderate**	**< 0.001**
2. During the past week, how much did the baby usually spit-up (anything coming out of the mouth) during a typical episode?	**-0.488**	**Moderate**	**< 0.001**
3. During the past week, how often did spitting up (anything coming out of the mouth) seem to be uncomfortable for the baby, for example, crying, fussing, irritability, etc.?	-0.296	Weak	< 0.001
4. During the past week, how often did the baby refuse a feeding even when hungry?	0.133	Very weak	< 0.001
5. During the past week, how often did the baby stop eating soon after starting even when hungry?	0.019	Very weak	ns
6. During the past week, did the baby cry a lot during or within 1 h after feedings?	-0.326	Weak	< 0.001
7. During the past week, did the baby cry or fuss more than usual?	-0.025	Very weak	ns
8. During the past week, on average how long did the baby cry or fuss during a 24 h period?	-0.293	Weak	< 0.001
9. During the past week, how often did the baby have hiccups?	**-0.593**	**Moderate**	**< 0.001**
10. During the past week, how often did the baby have episodes of arching back?	-0.337	Weak	< 0.001
11. During the past week, has the baby stopped breathing while awake or struggled to breathe?	-0.119	Very weak	< 0.001
12. During the past week, has the baby turned blue or purple?	-0.062	Very weak	0.081

Correlation between age and GER-related symptoms, as surveyed with the I-GERQ-R. Feeding refusal was the only symptom with a significant positive (very weak) correlation with age. The other correlations seen were negatively correlated with age. The strongest correlations seen (i.e. significant moderate correlations) are shown in boldface

### Regurgitation and crying symptoms over time

**Fig. 3:**
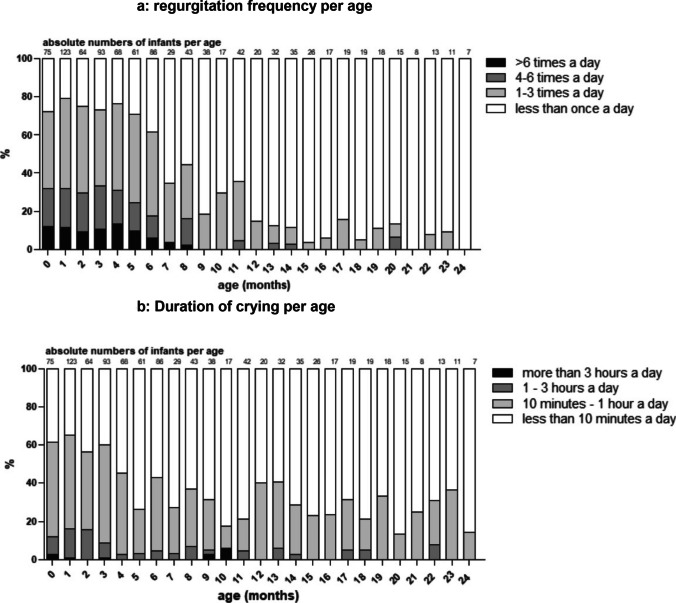
**a** Regurgitation frequency displayed as percentages of the four different categories of regurgitation frequency; **b** Crying frequency displayed as percentages of the four different categories of crying time a day

Regurgitation, for parents or caregivers defined as anything coming out of the mouth, was present in 70–79% of infants in the first 6 months of age (median 1–3 times a day) with a peak prevalence of 79% at the age of 1–2 months (Fig. 3a). Of infants aged 0–6 months (n = 570) old that regurgitate daily (n = 415, 73%), 251 (24%) regurgitated 1–3 times a day, 105 (18%) 3–6 times a day and 59 (10%) > 6 times a day.

Between 7–12 months old, prevalence of regurgitation ranged between 44 and 15% and dropped further to 0% afterwards.

Crying symptoms in infants decreased faster than regurgitation symptoms: infants aged 0–4 months cried the most (median crying times of 10 min up to an hour, Fig. 3b).

Fifty-five percent of infants aged 4 months and older had a reported crying time of less than 10 min a day.

### Association between regurgitation and colic subscores

The regurgitation subscore showed a significant weak correlation with colic-associated subscore of the I-GERQ-R (*r*_*s*_ = 0.393; *p* < 0.001).

### Association between regurgitation and feeding problems

The regurgitation subscore did not correlate with the frequency of feeding refusal (*r*_*s*_ = *-*0.049, *p* = 0.125).

## Discussion

To our knowledge, this is the largest study documenting age-dependent I-GERQ-R scores in a cohort of healthy infants aged 0–24 months. It shows that I-GERQ-R normal values are age-dependent and that up to 16% of young healthy infants have a score that is above the previously suggested cut-off score of 16 for GER*-*disease [4].

In this study [4], symptomatic patients might have been incorrectly attributed to the GER-disease group while in fact, their symptoms might have not been due to GER-disease [4].

Furthermore, by excluding symptomatic patients in the control group, the study did not evaluate the difference between patients *with- and without* GER-disease but the difference between subjects with and without symptoms [4, 7]. Last, age-related differences in the I-GERQ-R were not taken into account. These may all be reasons why previous attempts to validate the I-GERQ-R questionnaire for clinical use as a diagnostic tool failed.

Our results are in line with other, yet smaller studies with different study-populations [8, 9]. These studies reported I-GERQ-R scores > 16 in 26% and 19% of infants aged 1 month old [8, 9]. I-GERQ-R scores decreased with age and similar to our results, one of these studies showed that only 2% of infants aged 12 months had scores above the cut-off of ≥ 16 [9].

Overall, GER symptoms as measured with the I-GERQ-R, decreased significantly with age, mostly in the first 12 months of life. The strong decline in I-GERQ-R score in the first year of life was mainly caused by a reduction of regurgitation and crying symptoms and hiccup frequency. After the age of 1 year, these symptoms disappeared in nearly all infants.

After the age of 12 months, the overall I-GERQ-R score stabilized. While regurgitation prevalence further decreased, feeding refusal increased from the age of 15 months onwards. It should be noted that this concerns parent-reported feeding problems in a group of healthy subjects. It could thus well be that a developmental change in behavior in older infants and toddlers may have impacted feeding behavior, which was conceived to be problematic by parents. In fact, an increase in feeding problems after infancy was described by others as well and feeding problems in pre-school children are reported to occur in the normal population in up to 50% and are often considered part of normal development in toddlers [10, 11].

Regurgitation in infants is common and has, similar to our results, been shown to decrease significantly with age in multiple studies [8, 9, 12–17]. Due to the different definitions of regurgitation used in these studies, peak prevalence and peak age of regurgitation differed. Only one study assessed regurgitation symptoms beyond the age of 12 months and reported that symptoms diminished with age, with only 5% of infants ‘spilling more than half of their feeds’ at the age of 14 months [13].

The age-dependent *normal* values reported in this study should not be mistaken for *diagnostic* cut-off values to differentiate infants with and without GER-disease. Evaluation of I-GERQ-R values in a large cohort of patients with well characterized GER-disease is needed, after which validation as a diagnostic instrument should take place. While such studies may be difficult to perform, a validated age-dependent non-invasive tool to distinguish infants with GER-disease from those with symptoms related to physiological GER is highly needed.

The high outliers in our study show that the I-GERQ-R may not prove to be a perfect test to diagnose GER-disease after all, but even if it could be validated to rule out GER-disease with certainty, this would be a large step forward in reducing the overprescription of PPI [18–20].

The rapid decrease of the I-GERQ-R score in young infants should be taken into account not only for the further development of diagnostic cut off values for the presence or absence of GER-disease, but also be considered when interpreting results in therapeutic trials. Our study suggests that a decrease of 5 points, which is currently considered clinically relevant [4], has a different clinical relevance for young infants as compared to older ones and may also simply be attributed to increasing age.

Strengths of our study are the large number of subjects and the strict inclusion criteria to ensure that our cohort consists of healthy infants without GER-disease. To reduce selection bias, we visited well-baby clinics in urban neighborhoods with different socio-economic statuses. The well-baby clinics in the Netherlands are visited by nearly all healthy infants and toddlers in the country [21, 22].

Limitations of our study include the relatively low inclusion numbers of infants aged > 16 months. Additionally, we did not survey tobacco exposure and infant’s diets. Whether these parameters may potentially have an impact on GER symptoms, is still under debate [8, 13, 23–26].

## Conclusion

Gastroesophageal reflux symptoms measured by the I-GERQ-R, decrease in the first 24 months of age in healthy infants. Our results show that total scores of ≥ 16 should not necessarily be considered pathological in young infants. Future studies in infants with- and without GER-disease are needed to validate age-dependent I-GERQ-R cut-off values and to evaluate whether these can discriminate between infants with physiological GER and GER-disease.

### Supplementary Information

Below is the link to the electronic supplementary material.

Supplementary file1 (DOCX 14 KB)

## Abbreviations

GER: Gastroesophageal Reflux
GER-disease: Gastroesophageal reflux disease
I-GERQ-R: Infant Gastroesophageal Reflux Questionnaire Revised
