# Supplementary material for: Age-dependent normal values for the ‘Infant Gastroesophageal Reflux Questionnaire Revised’
Source: Eur J Pediatr. 2023 Nov 6;183(1):445–52. doi: 10.1007/s00431-023-05281-w (PMC10858078; doi:10.1007/s00431-023-05281-w)
Supplement: Supplementary file 1 — Supplementary file1 (DOCX 14 KB) [file 431_2023_5281_MOESM1_ESM.docx]

**Supplemental file 1: short clinical survey**

- What is your child's date of birth?
  - DD/MM/YYYY
- What is your child's gender?
  - Boy
  - Girl
- After how many weeks did you deliver your baby?
  - __ weeks + __days
- Have you ever consulted a health care provider for spitting up or crying?
  - Yes
  - No
